# Integrative inference of gene-regulatory networks in Escherichia coli using information theoretic concepts and sequence analysis

**DOI:** 10.1186/1752-0509-4-116

**Published:** 2010-08-18

**Authors:** Christoph Kaleta, Anna Göhler, Stefan Schuster, Knut Jahreis, Reinhard Guthke, Swetlana Nikolajewa

**Affiliations:** 1 Systems Biology/Bioinformatics Group, Leibniz Institute for Natural Product Research and Infection Biology - Hans Knöll Institute, Beutenbergstr. 11a, D-07745 Jena, Germany; 2 Dept. of Bioinformatics, Friedrich-Schiller-University Jena, Ernst-Abbe-Platz 2, D-07743 Jena, Germany; 3 Dept. of Genetics, University of Osnabrück, Barbarastraße 11, D-49076 Osnabrück, Germany

## Abstract

**Background:**

Although *Escherichia coli *is one of the best studied model organisms, a comprehensive understanding of its gene regulation is not yet achieved. There exist many approaches to reconstruct regulatory interaction networks from gene expression experiments. Mutual information based approaches are most useful for large-scale network inference.

**Results:**

We used a three-step approach in which we combined gene regulatory network inference based on directed information (DTI) and sequence analysis. DTI values were calculated on a set of gene expression profiles from 19 time course experiments extracted from the Many Microbes Microarray Database. Focusing on influences between pairs of genes in which one partner encodes a transcription factor (TF) we derived a network which contains 878 TF - gene interactions of which 166 are known according to RegulonDB. Afterward, we selected a subset of 109 interactions that could be confirmed by the presence of a phylogenetically conserved binding site of the respective regulator. By this second step, the fraction of known interactions increased from 19% to 60%. In the last step, we checked the 44 of the 109 interactions not yet included in RegulonDB for functional relationships between the regulator and the target and, thus, obtained ten TF - target gene interactions. Five of them concern the regulator LexA and have already been reported in the literature. The remaining five influences describe regulations by Fis (with two novel targets), PhdR, PhoP, and KdgR. For the validation of our approach, one of them, the regulation of lipoate synthase (LipA) by the pyruvate-sensing pyruvate dehydrogenate repressor (PdhR), was experimentally checked and confirmed.

**Conclusions:**

We predicted a set of five novel TF - target gene interactions in *E. coli*. One of them, the regulation of *lipA *by the transcriptional regulator PdhR was validated experimentally. Furthermore, we developed DTInfer, a new R-package for the inference of gene-regulatory networks from microarrays using directed information.

## Background

Gene regulation represents a central mechanism in the control of the phenotype of an organism. Thus, the comprehension of gene regulatory mechanisms is a central topic in Systems Biology [1]. The prokaryote *Escherichia coli *is best suited as a model organism for genome-wide network inference studies due to the available and well-documented molecular biological knowledge and the remarkable amount of published genome-wide data. Relevance or association networks [2] are widely used for genome-wide network inference. They require, first, a measure to evaluate association of pairs of genes, second, a threshold to cut off irrelevant associations, and, third, a criterion or algorithm to discriminate between direct and indirect interactions. The ready-to-use algorithms ARACNE [3,4], Context Likelihood of Relatedness (CLR, [5]) and MRNET [6] use mutual information (MI) as the association measure. A drawback of MI is represented by the fact that it is an undirected measure. That is, to derive causal relations from the inferred associations between interacting nodes, further information is necessary, in particular, to qualify one node as the regulator and the other as the target. There are several ways to derive a causal interaction from an inferred association: First, one can integrate prior knowledge. In [5] the inferred interactions are restricted to cases where one partner is a transcription factor (TF). Another approach is to use active and gene-specific interventions, like knockouts, knockdowns or over expressions. A third way is to exploit time series data and use them to infer the direction of association from temporal patterns. In this context directed information (DTI, [7]) can be used. DTI is an extension of the concept of MI that allows to measure the direction of an information flow between two random variables. It has been used earlier to infer gene regulatory mechanisms in kidney development [8]. In this work we improved the computation of DTI and used it to infer regulatory networks on a genome scale.

A second important step in the inference of gene-regulatory networks is the integration of additional knowledge. This process allows one to reduce the number of false positive predictions. One such approach is the integration of information extracted from genome sequence data. For predicted interactions between TFs and genes it is, for instance, possible to align the promoter regions of the predicted targets of a specific TF with each other to detect overrepresented motifs [5]. One possible explanation of such overrepresented motifs is that they correspond to a binding site of a common TF. On the other hand, if some binding sites of a TF are already known, the promoter regions of the putative target genes can be searched for sequences resembling these known binding sites. However, the sequences of binding sites can be very heterogeneous. In consequence, a binding site can be additionally validated by checking its phylogenetical conservation over several species [8]. This approach was used in this work.

A third step to reduce the number of false positive interactions is to integrate prior knowledge in form of known functional relationships between the regulator and the predicted target into the inference procedure. Finally, the predicted interactions have to be verified experimentally. In order to select a candidate interaction to verify we chose the regulation of a gene by a transcription factor whose targets are most suitably detected by our method. Thus, the present work demonstrates the full cycle of systems biological work, from genome-wide data analysis via large-scale modeling, to prediction of testable hypothesis by studying certain regulatory modules of interest, and, finally, to the prediction and experimental validation of novel molecular mechanisms.

## Results and Discussion

**Figure 1:**
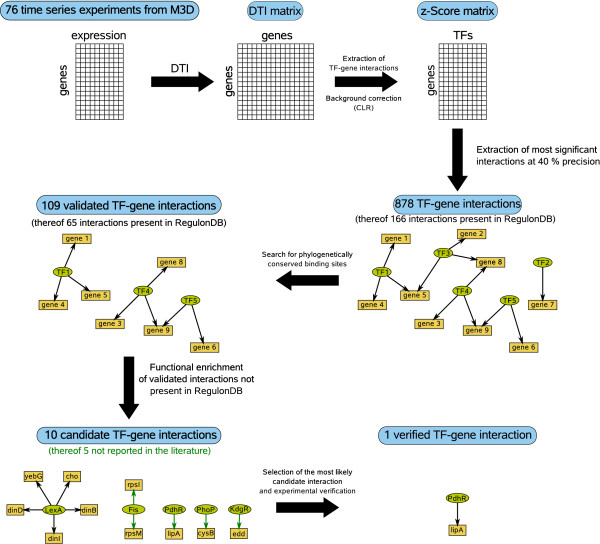
**Setup of the analysis**.

The setup of our analysis is outlined in Fig. 1. The procedure started by the extraction of microarray experiments containing equidistant time-series data from the Many Microbes Microarray Database (*M*^3*D*^, [9]). In the second step a DTI matrix was calculated. Afterward, TF-gene interactions were selected and a background correction was performed using the CLR algorithm [5] by computing the significances (*z*-scores) of the DTI values. The threshold for the acceptance of an interaction was determined by a comparison of the inferred network to known TF-gene interactions contained within RegulonDB version 6.1 [10]. There are 316 known and predicted TFs denoted in RegulonDB (see Additional File 1: Supplemental Material S6 for a list of TFs). A precision of 40% was used to calculate the threshold for the z-score. Usually, precision is defined as the fraction of known interactions within the inferred graph. Note that we did not compute here precision in this classical sense, but similar to [5](see *Methods*). Thus, we inferred 878 interactions, of which 166 are known. We compared our results to those that can be obtained using other inferences procedures building on mutual information. At the same precision, CLR based on mutual information [5] infers 1155 interactions of which 175 are known. Using ARACNE [3,4] and the same mutual information matrix we obtain 167 interactions of which 21 are known. In conclusion, our method, i.e. DTI with CLR, as well as mutual information in combination with CLR perform much better than ARACNE that does not use CLR. For a more detailed comparison of precision at different numbers of inferred interactions see Additional File 1: Supplemental Material S3.

The inferred interactions were validated by a search for a phylogenetically conserved binding site of the TF upstream of its putative target. The validated interactions were manually enriched for functional relationships between the regulator and the targets to select candidates for an experimental verification. Finally, the most promising regulatory interaction between PdhR and *lip*A was experimentally verified by an electrophoretic mobility shift assay (EMSA).

### Validation of predicted interactions by sequence analysis

The predicted TF - gene interactions were validated by searching for phylogenetically conserved transcription factor binding sites (TFBS) in the promoter region of the presumed target genes. To this end known binding sites of the regulator are aligned with the promoter region of the putative target using the motif discovery tools *cosmo *[11]. If a region that resembles the known binding sites of the regulator was discovered, we checked whether this region overlaps to more than 50% with a phylogenetically conserved region of the genome. If we found such an overlap, the interaction was accepted. For more information on the search for binding sites see *Methods *and Additional File 1: Supplemental Material S4. This leads to 109 accepted interactions, of which 65 are known according to RegulonDB [Additional File 1: Supplemental Material S7]. While the total number of predicted interactions dropped from 878 to 109, the fraction of known interactions increased from 166/878 = 19% within the network inferred using DTI to 65/109 = 60% when additionally requiring the presence of a phylogenetically conserved binding site of the regulator. Thus, the search for phylogenetically conserved binding sites reduces the number of inferred interactions to a much smaller set which is supported by additional evidence. However, due to this step we might also loose true positive interactions since only about one third of the TFBS overlap to more than 50% with a conserved region of the *E. coli *genome [Additional File 1: Supplemental Material S4].

For the 44 (109-65) interactions not reported in RegulonDB for which we found a phylogenetically conserved binding site, we checked for functional relationships between the regulator and the target. Thus, we found ten interactions (table 1). Five of them have already been reported in the literature [12-14], but were not yet included in RegulonDB 6.1.

**Table 1:** Predicted regulatory interactions

TF	Target	*z***-score**	TFBS conservation	**Ind. Ev**.
LexA	*cho*	9.45	68.6%	[12,16]
	*dinB*	8.38		[12,16]
	*dinI*	9.84		[12,16]
	*dinD*	7.69		[12,16]
	*yebG*	10.39		[12,16]

Fis	*rplM ^$^*	-	15.1%	-
	*rpsI*	6.59		

PdhR	*lipA*	9.42	72.9%	this study

PhoP	*cysB*	7.6	21.7%	-

KdgR	*edd*	8.92	0%	-

Overview over the regulatory interactions inferred by the use of DTI and validated through a phylogenetically conserved TFBS in the promoter region of the target gene not yet reported in RegulonDB. A total of 10 interactions of which five have already been reported elsewhere are predicted (indicated in column five). Of the 44 interactions that have been not included in Regulon DB v. 6.1 but have been validated through the search for a phylogenetically conserved binding site of the regulator, ten were ascertained through a functional relationship of the putative target with other known targets of the regulator. In the third column the *z*-score of the interaction and in the forth column the average overlap of known TFBS of the regulator with conserved regions of the genome are given. The interaction marked with ^$ ^lies within the same operon like a gene for which an interaction has been inferred. For additional interactions validated through the chromosomal location of the target downstream of an operon known to be regulated by the putative regulator see Additional File 1: Supplemental Material S8.

### Predicted and functionally related interactions

#### Predicted targets of LexA

**Figure 2:**
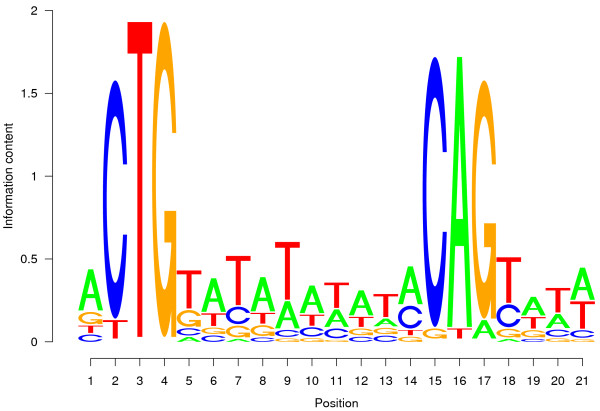
**Sequence logo of the position weight matrix of the LexA binding site**. The sequence logo graphically represents the consensus sequence of the binding site, with the height of each letter indicating the information content of each position.

LexA is an important regulator in the bacterial SOS response allowing to bypass lesions or errors in DNA during replication [15]. As already observed by [5], many of the known targets of LexA can be correctly identified from microarray experiments present in M^3*D*^ since a significant portion of the experiments stored in this database involve DNA damage. Additionally, LexA binds to a very specific DNA sequence (Fig. 2): the LexA binding site is flanked by three very well preserved nucleotides containing a stretch of AT repeats.

The search for phylogenetically conserved binding sites for predicted interactions identified five new targets of the transcription factor LexA: *cho*, *din*B, *din*I, *din*D and *yebG*. These five genes have been reported previously to be regulated by LexA [12,16], but were not yet included in Regulon DB 6.1.

#### PdhR

We found strong evidence, both on an expression and phylogenetic level, for the regulation of *lipA *by PdhR. PdhR is an important regulator of central metabolism by controlling the transcription of the components of the pyruvate dehydrogenase complex and several genes involved in the respiratory chain [17]. *LipA *encodes the lipoate synthase which catalyzes the last step in lipoate biosynthesis and incorporation. Lipoate is an important co-factor of LpdA that is contained in the the pyruvate dehydrogenase complex, oxoglutarate dehydrogenase and the glycine cleavage complex [18].

#### PhoP

According to RegulonDB, PhoP binds in the promoter regions of 31 genes. Among the genes regulated by PhoP are two genes involved in methionine biosynthesis. One of the corresponding enzymes, encoded by *metB*, catalyzes the step of the incorporation of sulfur contained within cysteine into O-succinyl-L-homoserine to produce cystathionine, subsequently converted into methionine. A putative phylogenetically conserved binding site of PhoP in the upstream region of *cysB *was detected. *CysB *encodes a TF regulating several genes necessary for the production of cysteine from which methionine is synthesized in *E*. *coli *[19].

#### KdgR

A newly predicted target of KdgR is *edd *encoding a gluconate dehydratase in the Entner-Doudoroff pathway. While a binding site of KdgR in the upstream region of *eda*, the Entner-Doudoroff aldolase, is known [20], hitherto no binding site upstream of *edd *which precedes *eda *on the chromosome has been reported. Regulation by KdgR induces *eda *if glucuronate, galacturonate, or methyl-*β*-d-glucuronide are present in the growth media [20]. The activation of *eda *allows the growth on these compounds. The existence of a binding site upstream of *edd *would furthermore allow a control of the metabolic flux into the pentose-phosphate-pathway.

#### Fis

**Figure 3:**
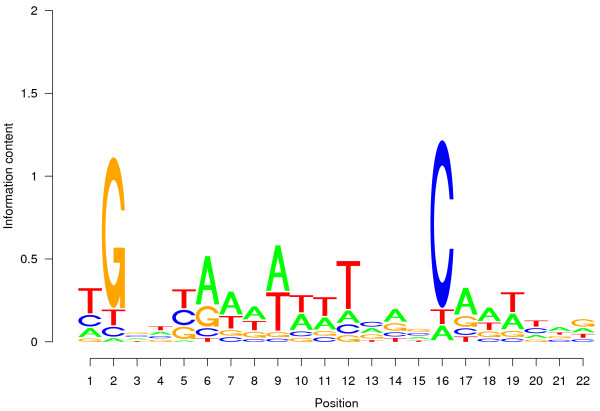
**Sequence logo of the position weight matrix of the Fis binding site**. In contrast to the LexA binding site (Fig. 2) only few positions are preserved.

Fis is a small protein that plays an important role in the organization and maintenance of nucleotide structure by binding to DNA. Furthermore, it modulates the expression of other proteins serving this purpose [14,21] and is involved in the regulation of many other processes of the cell. In [14] it was found that the expression of 21% of all genes of *E*. *coli *changed after an knockout of Fis. Due to its involvement in nucleotide organization, only some of the binding sites of Fis confer a regulatory influence. In contrast to LexA, the binding sites of Fis are less preserved in their sequence (Fig. 3). In consequence, the evidence from a detected binding site of Fis in the promoter regions of *rplM *and *rpsI *is not as strong as in the case of the detected binding sites of LexA. *rplM *and *rpsI *encode ribosomal proteins. Many proteins of the translational apparatus in turn are known to be regulated by Fis [22]. However, in [14] who studied the distribution of Fis binding sites within the entire genome of *E. coli*, no binding site of Fis upstream of these two genes was identified.

### Selection of candidates and experimental verification

In order to select a candidate for the experimental validation of a predicted interaction we compared the *z*-scores and the average phylogenetical conservation of known binding sites of each TF (Table 1) for the interactions reported in the last section. Especially, the TFBS of LexA and PdhR are well conserved. Furthermore, the *z*-scores of the interactions of these regulators are the highest. Since the predicted targets of LexA have already been experimentally verified in [12,16], we thus chose the regulation of *lip*A by PdhR as best candidate for an experimental validation of a predicted interaction.

**Figure 4:**
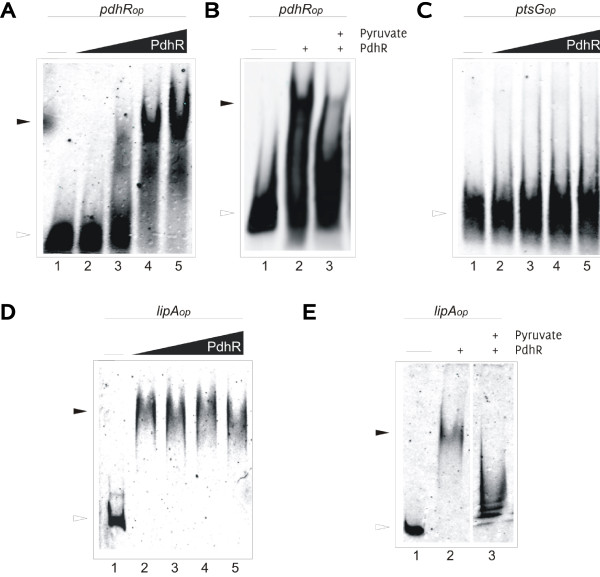
**Gel shift assay**. Fluorescently labeled DNA probes were incubated with purified PdhR protein. All samples were directly loaded onto a polyacrylamide gel after incubation. Arrows indicate the free, filled arrows the shifted DNA, respectively. **A **The PdhR binding site of the *pdhR-aceEF-ldpA *operon was incubated with increasing amounts of PdhR as indicated (9, 18, 28, 37 pmol). **B **The PdhR binding site was incubated with 39 pmol PdhR (lane 2) and with PdhR and 50 mM pyruvate (lane 3). **C **The Mlc consensus sequence was incubated with purified PdhR (18, 28, 37, 46 pmol). **D **The operator promoter sequence of *lipA *(ca 500 bp in front of the start codon) was incubated with PdhR (33, 44, 50, 55 pmol) **E **The *lipA *operator promoter was incubated with 44 pmol PdhR (lane 2) and with PdhR and 100 mM pyruvate (lane 3).

To confirm the presence of a PdhR binding site in the promoter region of *lipA*, we performed electrophoretic mobility shift assays. As a positive control we used standard conditions to detect the PdhR-dependent shift of the DNA fragment of the previously reported PdhR binding site within the promoter region of the *pdhR*-*aceEF*-*ldp*A operon (Fig. 4A). Previous studies on the regulation of PdhR unveiled that pyruvate inactivates the PdhR binding activity in several promoter regions including the PdhR binding site in the *pdhR-aceEF-ldpA *operon [17]. We could confirm this result, which also indicated a completely functional PdhR protein (Fig. 4B). To exclude unspecific binding of PdhR to heterologous DNA, which does not contain any PdhR binding site, we incubated the repressor with a DNA fragment, which includes the Mlc binding site within the *ptsG *operator promoter region. No shift of this DNA fragment was observed (Fig. 4C). For the promotor region of *lipA*, the PdhR-dependent complex of DNA and repressor protein became evident in the appearance of the shifted promoter region of *lipA *(Fig. 4D). Furthermore, we could demonstrate that the interaction of PdhR with the *lipA *promoter region was abolished in the presence of pyruvate (Fig. 4E).

### R-package for Network Inference

The methods for the inference of gene regulatory networks presented in this work have been implemented in an R-package that can be downloaded from http://users.minet.uni-jena.de/~m3kach/DTInfer/[Additional File 2]. Given the time-courses of expression values for a set of genes over one or several experiments, DTI values are computed between arbitrary sets of genes. Additionally it is possible to compute MI values. DTIs and MIs can be estimated using one of two estimators; a kernel density estimator we implemented based on the work of [23] and a b-spline estimator based on the work of [24], implemented by Boris Hayete and provided by courtesy of the Gardner Lab of the Berkeley University. Significance of the MI or DTI values, as well as those provided by the user are assessed through the Context Likelihood of Relatedness algorithm presented in [5]. Finally, interactions are inferred either at a certain precision (see *Methods*), for a given number of interactions or a user-defined threshold.

## Conclusions

In this work we used directed information (DTI) to infer transcription factor gene interactions in *E. coli*. In contrast to previous works using DTI [8] we improved the inference procedure in several points. First, we used a more precise algorithm for the computation of mutual information required for the estimation of DTI. Second, we used CLR [5] in order to determine the significance of the DTI values. This step is necessary to remove interactions starting or ending in genes which have high DTI values with many other genes. Third, we validated the inferred interactions by the search for phylogenetically conserved transcription factor binding sites. Especially this last step allows to drastically increase the fraction of true positives in the set of inferred interactions. Finally, by additionally requiring a functional relationship between regulator and target, we extracted a set of ten TF - gene interactions of which five are unknown in the literature. We predicted that PhoP regulates *cysB *encoding a global regulator of cysteine biosynthesis, KdgR putatively regulates *edd *encoding the gluconate dehydratase, Fis putatively regulates *rpsI *and *rplM *encoding two ribosomal proteins, and PdhR regulates *lipA *encoding the lipoate synthase.

Experimentally validating the most likely candidate of a predicted interaction we were able to shed new light on the regulation of central metabolism. We found that the transcription factor PdhR does not only regulate the expression of the the pyruvate dehydrogenase (PDH) multi-enzyme complex, but additionally controls the production of the co-factor lipoate required for the activity of this enzyme complex by regulating the expression of the lipoate synthase LipA. Thus, these new findings further emphasize the role of pyruvate-sensing PdhR in the control of the activity of LpdA, the E3 component of the pyruvate dehydrogenase complex, the oxoglutarate dehydrogenase complex and the glycine cleavage complex. Moreover they underline the key role of the regulator PdhR in the control of fluxes at the pyruvate node that connects glycolysis, citric acid cycle and lipid metabolism.

Additional to the ten predicted interactions, we found eight cases for which we did not detect a phylogenetically conserved binding site, but we could support our prediction using data from the literature [Additional File 1: Supplemental Materials S8 and S9]. In one case a binding site has been detected independently. In seven cases an alternative operon structure reported in the literature supports the predicted interactions.

In conclusion, our work demonstrates the importance of integration of different types of data and prior knowledge into network inference algorithms in order to stringently plan new experiments that are able to identify hitherto unknown molecular interactions in gene regulatory networks. We started from a large compendium of gene-expression experiments and inferred 878 putative regulatory interactions. By probing these predicted interactions with independent knowledge from phylogenetic and sequence data we were able to narrow down the list of potential interactions to a smaller list of 109 validated interactions, which could be surveyed by manual inspection. Of the 44 interactions contained in this list, which were not yet present in RegulonDB 6.1, we identified ten interactions where we could also identify a functional relationship between the regulator and the target. Of these ten targets, five were already reported in the literature. Thus, we narrowed down the list of 878 interactions to five very likely targets that should be verified by experiment. Finally, genome-wide data analysis and modeling was the driving force to design experiments for the discovery of the regulation of *lipA *by PdhR. In consequence, our approach further emphasizes the vital importance of the combination of different bioinformatics methods for saving resources in experimental work by in silico selection of most likely candidates for the time-consuming and expensive procedure of experimental verification.

## Methods

### Directed information

Directed information can be interpreted as a directed version of mutual information [7] that allows to measure the information flow between the time-series of the expression of two genes. Given two random processes *X *and *Y *of length *N*, the DTI *I*(*X^N^*→*Y^_n_^*) is defined as

(1) $$I\left(X^{N}\to Y^{N}\right)=\sum_{n=2}^{N}I\left(X^{n};Y_{N}|Y^{n-1}\right)$$

where *Y^n ^*denotes (*Y*_1_,*Y*_2_, ..., *Y*_n_), that is, a segment of the realization of the random sequence *Y*. DTI can be interpreted as the mutual information between the time course of *X *to the current point *n *and the current value of *Y *given all values of *Y *up to the previous instant *n *- 1. Since we are summing over all time-points we are taking into account the relationship for every time point.

Equation 1 contains a conditional term, hence it can be reformulated using the relationship *I*(*X^N^,Y^N^*) = *H*(*X^N^*) - *H*(*X^N^*|*Y^N^*) between mutual information *I*(··;··), entropy *H*(·) and conditional entropy H(·|·)[8]

$$\begin{array}{c} \;I\left(X^{N}\to Y^{N}\right)\;=\sum_{n=2}^{N}\left(H\left(X^{n}|Y^{n}{}^{-1}\right)\;-H\left(X^{n}|Y^{n}\right)\right)\;\\ =\sum_{n=2}^{N}\left((-H\left(X^{n}|Y^{n}\right)\;-\left(\;-H\left(X^{n}|Y^{n}{}^{-1}\right)\right)\right)\\ =\sum_{n=2}^{N}(H\left(X^{n}\right)-H\left(X^{n}\right)-H\left(X^{n}|Y^{n}\right)\;\\ \;\;-\left(-\;H\left(X^{n}|Y^{n}{}^{-1}\right)\right))\\ =\sum_{n=2}^{N}(H\left(X^{n}\right)\;-H\left(X^{n}|Y^{n}\right)\\ \;-\left(H\left(X^{n}\right)\;-H\left(X^{n}|Y^{n}{}^{-1}\right)\right))\\ =\sum_{n=2}^{N}\left(I\left(X^{n};\;Y^{n}\right)\;-I\left(X^{n};\;0Y^{n}{}^{-1}\right)\right) \end{array}$$

where 0*Y^n-1 ^*denotes the concatenation of 0 and *Y*^n-1^, *i.e.*, (0,*Y*_1,_*Y*_2_,...,*Y*_n-1_). This concatenation is equal to considering pairs of (*X*^2^,*Y*^1^), (*X*^3^,*Y*^2^),...,(*X*^n^,*Y*^n-1^) of expression values in which the *X*-values are shifted one time-step into the future. In consequence, directed information can also be understood as the mutual information between *X *and *Y *subtracted by the information flow between the time series of *Y *shifted one step and *X*. Hence, by subtracting the causal (shifted) relationship from *Y *to *X*, the causal dependency from *X *to *Y *remains.

To evaluate the mutual information term in equation (2), a b-spline estimator based on the work of [24] and implemented by Boris Hayete of the Gardner Lab as part of the CLR algorithm has been used. More details on the implementation of the DTI estimator can be found in Additional File 1: Supplemental Material S1.

### Context Likelihood of Relatedness (CLR)

Having computed a DTI value, the significance of the value needs to be determined. That is, the probability that the DTI indicates a true dependency is to be assessed. The complementary event, the null-hypothesis, is represented by a DTI value that can be obtained from the expression series of randomly chosen non-interacting genes. The null-distribution of the DTI-values for a given context, *i.e.*, the distribution of DTI values for two independent genes, are not known. Hence they need to be estimated.

A method to perform this estimation is represented by the context likelihood of relatedness (CLR) algorithm [5]. CLR is an extension of the relevance networks approach [2] and has first been proposed by [4] for cluster-analysis. This approach makes explicit use of the data to estimate the null-distribution. The assumption underlying the approach is that there is no interaction between most gene pairs. Hence, the null-distribution of the DTIs can be obtained from the whole set of DTIs determined from a potential regulator to all other genes.

Furthermore, when using CLR, we do not consider only the value of the DTI within the set of potential regulators of a target gene. Thus, two *z*-scores are computed. The first is the *z*-score of the DTI within the null-distribution of DTIs for all potential targets of a regulator and the second the *z*-score of the DTI within the null-distribution of all potential regulators of a target gene. A cumulative *z*-score is computed as the quadratic mean of both *z*-scores. For TF-gene interactions, these *z*-scores are computed only within the matrix of TF-gene interactions. [5] in contrast computed *z*-scores within the full MI matrix and then extracted all those concerning interactions where one partner is a TF. More details on the implementation of the CLR algorithm are given in Additional File 1: Supplemental Material S2.

An interaction is accepted if the cumulative *z*-score is above a certain threshold. Similar to [5] this threshold is determined using precision, which is defined as the fraction of known interactions within the set of inferred interactions. However, since not all TFs and genes are equally well studied, we use only a subsystem of the inferred network that contains genes having known regulators or TFs having known targets as a reference. Thus, for the computation of precision, edges corresponding to TFs or genes without known targets or regulators, respectively, are removed. Then, we determine the known interactions by a comparison of inferred TF - gene interactions to the interactions contained within RegulonDB. Finally, we compute precision as the number of known interactions divided by the number of inferred interactions in the reduced graph.

### Sequence-based validation of TF - gene interactions

Inferred interactions are validated through independent evidence. This process helps to reduce the number of detected interactions to a smaller set containing a higher fraction of true interactions. A direct approach is to search for putative binding sites of the regulator in the promoter region of the target gene. This process is separated into two steps. First, a putative binding site of the TF is searched in the promoter region of the target gene. Then, this binding site is checked for phylogenetical conservation.

The discovery of binding sites can be performed using various approaches [11,25,26]. Here, the R-package *cosmo *[11] was used. *cosmo *allows us to detect overrepresented motifs in DNA sequences. Binding sites are detected by passing known binding sites of the TF along with a stretch of 400 base pairs upstream of the start site of the presumed target gene to *cosmo *(for more details on the detection of binding sites see Additional File 1: Supplemental Material S4).

In order to validate the predicted binding site its phylogenetical conservation over different species is checked. Phylogenetically conserved regions upstream of genes in ten proteobacterial genomes have been identified in [27,28]. The assumption that underlies this analysis is that TFBSs are under a positive selective pressure and hence can be identified by comparing stretches of upstream regions of orthologous genes in several species. If a conserved region overlaps to more than 50% with a putative binding site, the interaction is accepted.

### Data

**Figure 5:**
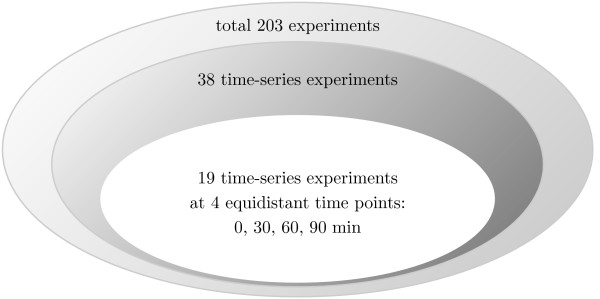
**Types of experiments in M^3*D*^**. M^3*D *^contains 630 uniformly normalized Affymetrix chips for *E. coli *[5]. For the computation of DTI 88 microarray experiments from 19 experiments over the same time interval of 30 min were used.

Gene expression data has been obtained from the dataset E_-_coli_-_v4_-_Build_-_4 of the Many Microbe Microarray Database [9] released in December 2007. Of the 203 experiments, 38 contain time-course data. Since DTI requires equidistant time data, experiments for which data of 0, 30, 60 and 90 minutes are available were chosen (Fig. 5). In some cases data for 120 and 180 minutes is additionally available. However, for a time-span of 90 minutes, M^3*D*^contains 19 experiments, which is larger than for other time spans. Using for example data stretching over 120 minutes, fewer true positive interactions are inferred when comparing to the use of data stretching over 90 minutes (not shown). In some cases, where time-point 90 minutes is missing, but 120 minutes available, 90 minutes was estimated using a spline interpolation [29]. An overview on the experiments used and the time-points available is given in table 2. Data on known TF gene interactions, a list of known and predicted TFs, position within the chromosome and arrangement within operons were collected from RegulonDB release 6.1 [10]. Information on the function of gene products was obtained from EcoCyc [30].

**Table 2:** List of experiments

ID	Time points (min)	Replicates
*ccdB overexpression, o-phenanthroline chelator, recA knockout, different E. coli strains*		
*cc*dB_-_K12	0, 30, 60, 90, 120	1
*cc*dB_-_MG1063	0, 30, 60, 90, 120	2
*cc*dB_-_W1872	0, 30, 60, 90	1
*cc*dB_-_chelator W1872*	0, 30, 60, 120	1
*cc*dB_-_chelator MG1063*	0, 30, 60, 120	1
*cc*dB_-_BW25113*	0, 30, 60, 120, 180	1
*cc*dB_-_MG1655	0, 30, 60, 90	2
*cc*dB_-_BW25113recA*	0, 30, 60, 120, 180	1
*lacZ up-regulation after induction, different E. coli strains*		
lacZ_-_K12	0, 30, 60, 90, 120	1
lacZ_-_MG1063	0, 30, 60, 90	2
lacZ_-_W1863	0, 30, 60, 90	1
lacZ_-_MG1655	0, 30, 60, 90	1
*norfloxacin, recA knockout, different E. coli strains*		
MG1063_-_uninduced*	0, 30, 60, 120	1
nor oxacin_-_MG1063*	0, 30, 60, 120	1
BW25113_-_uninduced*	0, 30, 60, 120, 180	1
norfloxacin_-_BW25113*	0, 30, 60, 120, 180	1
BW25113recA_-_uninduced*	0, 30, 60, 120, 180	1
norfloxacin_-_BW25113recA*	0, 30, 60, 120, 180	1
MG1655_-_uninduced	0, 30, 60, 90	1

Microarray data from M^3*D*^used for the inference of gene regulatory networks. Details on the experimental conditions can be obtained from M^3*D*^. Please note that for each experiment, only time-points 0, 30, 60 and 90 minutes were used. If time-point 90 minutes was missing, it was interpolated using splines (experiments marked with*).

### Experimental procedures

#### Plasmid construction

The *pdhR *gene was amplified by standard PCR with a pair of primers, PdhR+ (3'-CTGCAGGAACTCATGGCCTACAG-5') and PdhRhis- (3'-GAATTCCTAGTGGTGGTGGTGGT GATTCTTTCGTTGCTCCAG-5'). The latter encodes a C-terminal Penta-His-tag fused to the *pdhR *gene. Genomic DNA of the *Escherichia coli *K-12 derivative LJ110 [31] was used as template. The 797 bp PCR product was purified with DNA Purification System (Promega), ligated into the pGEM^®^-T vector (Promega) and sequenced (Scientific Research and Development GmbH). Via a 5' *Pst*Irestriction site provided by the primer PdhR+ and a 3' *Pst*Irestriction site provided by the pGEM^®^-T vector, the *pdhR-his *gene was cloned into the expression plasmid pTM30 [32] yielding pTM30PdhRhis.

#### Purification of His-tagged PdhR protein

His-tagged PdhR was overexpressed in *E*. *coli *JM109 [33] using the expression plasmid pTM30PdhRhis and purified using affinity chromatography as described previously [34]. Except that, for purification, frozen cells were resuspended in lysis buffer (50 mM Tris-HCl, pH 8.0 at 4°C, 100 mM NaCl) with 0.25 mM AEBSF and 0.25 mg/ml lysozyme. Other than Ni(II)-NTA agarose suspension was used, the supernatant was loaded onto a HisTrapTMFF column (GE Healthcare) and purified with the ÄKTA FPLC (GE Healthcare). The column was subsequently washed with 10 ml buffer N (20 mM Tris-HCl, pH 8.0 at 4°C, 0.1 mM EDTA, 500 mM NaCl, 5 mM 2-mercaptoethanol, and 5% glycerol) containing 5 mM imidazole, and with 20 ml buffer N containing 20 mM imidazole. The protein was eluted with buffer N containing 150 mM imidazole and the fraction containing his-tagged PdhR was dialyzed against storage buffer (50 mM Tris-HCl, pH 7.6 at 4°C, 200 mM KCl, 10 mM MgCl2, 0.1 mM EDTA, 1 mM DTT, and 50% glycerol). The protein concentration was determined with the Qubit fluorometer (Invitrogen) and the purity was checked by SDS-PAGE, western blot analysis and silverstain.

#### Gel shift assay

DNA probes were either generated by annealing equimolar amounts of fluorescence labeled primers (Thermo Fisher Scientific) of the PdhR-binding site (5'DY682-GCCGAAGTCAATTGGTCTTAC CAATTTCATGTCTGTG-3'and 5'DY782-CACAGACATGAAATTGGTAAGACCAATTGACTT CGGC-3') or the Mlc-binding site (5'DY782-TTGGCAAATTATTTTACTCTGTGTAATAAATAAA GGGCG-3' and 5'DY682-CGCCCTTTATTTATTACACAGAGTAAAATAATTCAGTGCCAA-3'). The promotor region (507 bp from the initiation codon) of *lipA *was amplified by PCR with fluorescence labeled primers (5'DY682-ACTATCGACAACGCTGCGCATG-3' and 5'DY782-TAGCGTGCGTGTTCCAGTT GCG-3'). The PCR product was purified with the DNA Purification System (Promega). The gel shift assays were performed as described previously [17] except that 0.1 pmol labeled DNA probe was added to the binding reaction. The PCR product of the *lipA *promotor region was used in a dilution of 0.025 pmol per reaction. The binding buffer and conditions were used as in [17], but 0.1 mg/ml buffer of heterologous herring sperma DNA was added. After addition of 5 *μ*l 50% glycerol to the binding reaction, the sample was loaded onto a 6% polyacrylamide gel. After gel electrophoresis the labeled DNA was detected by the Odyssey Scanner (Licor).

## Authors' contributions

CK and SN performed the analysis. CK wrote the R package for the inference of gene-regulatory networks using DTI. AG performed the experiments. SN, KJ, RG and SS advised, organized and guided the present study. CK, RG and SN drafted the manuscript. All authors read and approved the final manuscript.

## Supplementary Material

Additional file 1 **Supplementary Material**. Supplemental Material containing further information on the inference procedure and the inferred interactions. Click here for file

Additional file 2 **DTInfer**. R-package for the inference of gene-regulatory networks from microarray data using directed information and mutual information Click here for file
