# Supplementary material for: Integrative inference of gene-regulatory networks in Escherichia coli using information theoretic concepts and sequence analysis
Source: BMC Syst Biol. 2010 Aug 18;4:116. doi: 10.1186/1752-0509-4-116 (PMC2936295; doi:10.1186/1752-0509-4-116)
Supplement: Additional file 1 — Supplementary Material. Supplemental Material containing further information on the inference procedure and the inferred interactions. [file 1752-0509-4-116-S1.PDF]

Integrative inference of gene-regulatory networks in *Escherichia coli* using information theoretic concepts and sequence analysis

Christoph Kaleta[1,2,*], Anna Göhler[3], Stefan Schuster[2], Knut Jahreis[3], Reinhard Guthke[1] and Swetlana Nikolajewa[1]

[1] Systems Biology/Bioinformatics Group, Leibniz Institute for Natural Product Research and Infection Biology – Hans Knöll Institute, Beutenbergstr. 11a, D-07745 Jena, Germany
[2] Dept. of Bioinformatics, Friedrich-Schiller-University Jena, Ernst-Abbe-Platz 2, D-07743 Jena, Germany
[3] Dept. of Genetics, University of Osnabrück, Barbarastraße 11, D-49076 Osnabrück, Germany

S1 Directed information

The quality of the estimation of directed information depends on two important factors.

First, is the availability of sample replicates which can greatly enhance the estimation of the mutual information terms in equation 2 (main document) and thus the estimation of the DTI [1]. Sample replicates can be utilized using a direct approach. Given a time series of length N from r experiments for gene 1 and gene 2, $\mathbf{e}^1 = \mathbf{e}^1_{ij}$ is the matrix of measured expression values for experiments $1 \leq i \leq r$ at time-points $1 \leq j \leq N$. e^2_{ij} is the respective value for gene 2. Thus, the expression values are given by two $N \times r$ matrices $\mathbf{e}^1$ and $\mathbf{e}^2$ for gene 1 and gene 2 respectively. Then by replacing each X_n by $(e^1_{1n}, .., e^1_{rn})$ and X^n by $(X_1, X_2, .., X_n) = (e^1_{11}, e^1_{21}, .., e^1_{r1}, e^1_{12}, .., e^1_{r2}, .., e^1_{rn})$ and Y by the corresponding values in $\mathbf{e}^2$ in equation 2 (main document), $I(\mathbf{e}^1 \rightarrow \mathbf{e}^2)$ can be calculated as the DTI over several experiments.

Second, the type of mutual information estimator used is of importance. While [1] used an improved binning approach that estimates mutual information through adaptive binning, [2] used a b-spline based estimator presented in [3]. Tests on sample data (not shown), demonstrated an improved performance of the later estimator which was also used in this work. For the computation of DTI, 6 bins and a spline order of $k = 3$ were used (see [3] for details).

S2 Context of Likelihood Relatedness

The CLR algorithm, presented in [2] estimates the significance of an interaction by taking into account the network context of the regulator and the target gene. This is done by calculating a cumulative z-score or standard score as follows. For a set of n regulators, i.e., TFs, with m potential target genes, the $n \times m$ matrix $\mathbf{M}$ contains the computed DTIs for all possible interactions. An entry M_{ij} represents the DTI of the expression values of gene i to gene j. Now we can compute $z^{(1)}_{ij}$ as

*Corresponding author

$$z_{ij}^{(1)} = \frac{M_{ij} - mean(M_{i\cdot})}{std(M_{i\cdot})} \tag{1}$$

where $mean(M_{i\cdot})$ denotes the mean and $std(M_{i\cdot})$ the standard deviation of the DTIs in row i of $\mathbf{M}$ (i.e., for regulator i). Here the relative position of the DTI $I(\text{gene } i \rightarrow \text{gene } j)$ within the distribution of the DTIs of all potential targets of gene i is determined (if a normal distribution for the null distribution of the DTIs is assumed). In consequence, a high z_{ij}^{1} corresponds to a DTI significantly above the null-distribution of the DTIs.

Similarly, we can compute $z_{ij}^{(2)}$ as

$$z_{ij}^{(2)} = \frac{M_{ij} - mean(M_{\cdot j})}{std(M_{\cdot j})} \tag{2}$$

Here we determine the relative position of $I(\text{gene } i \rightarrow \text{gene } j)$ within the distribution of the DTIs of all potential regulators of gene j. [2] computed $\mathbf{z}^{(2)}$ over all genes. However, using DTI a higher number of true positives are found, if $\mathbf{z}^{(2)}$ is computed only over known or predicted TFs[1]. A cumulative z-score is then computed as

$$z_{ij} = \sqrt{{z_{ij}^{(1)}}^2 + {z_{ij}^{(2)}}^2} \tag{3}$$

Thus, z_{ij} takes into account the significance of M_{ij} within the potential set of genes regulated by gene i and the set of potential regulators of gene j.

S3 Comparison to other information theoretic approaches

We compared our method to two other methods for network inference using information theoretic measures: CLR [2] and ARACNE [4, 5] using mutual information. In Fig. 1 the precision, as defined in the main document, is displayed for different numbers of inferred interactions. In each case interactions were inferred using the same procedure as in the main document by starting with the interaction with the highest score, then the interaction with the second highest score and so on. As can be seen in Fig. 1 our approach (DTI+CLR) outperforms ARACNE (MI+ARACNE) but is less accurate than CLR in combination with mutual information (MI+CLR) which has on average 10% higher precision at the same number of inferred interactions.

S4 Validation of interactions

Inferred interactions are validated by searching for phylogenetically conserved binding sites of the TF in the promoter region of the target gene. Details on the discovery of putative binding sites using *cosmo* [6] are given in the following.

Detection of binding sites

The assumptions underlying the motif discovery in *cosmo* is that sequences are generated through a multinomial mixture model. This model assumes that a certain nucleotide is generated by a background model of the complete sequence or by a specific model describing the motif to be searched. The background model can be estimated either from the input sequences or from a user-defined set of DNA sequences. The background model has been estimated from intergenic

[1]Please note that this is only the case where TF - gene interactions are examined

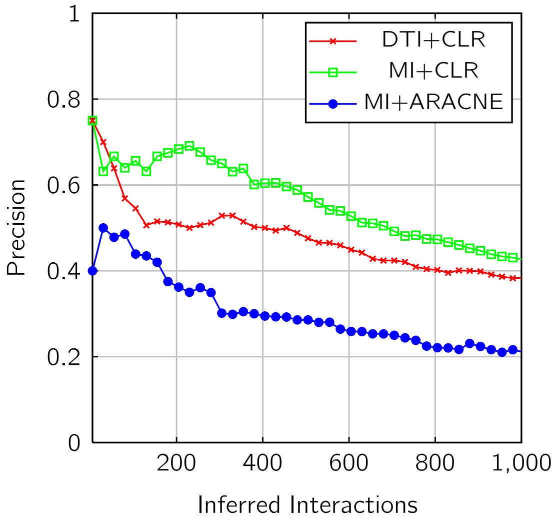

Figure 1: Comparison of information theoretic network inference procedures. On the x-axis the number of inferred interactions and on the y-axis the precision for this particular number of inferred interactions is given.

regions obtained from the EcoGene database (`ecogene.org`, [7]). Next, a motif model is estimated by searching regions in the DNA that significantly differ from the background model and appear in a specific subset of sequences. This subset can be defined by several approaches. The basic approach, "one-occurrence-per-sequence" (OOPS), tries to find a motif in each of the given sequences. Alternatively, the "zero-or-one-occurrence-per-sequence" (ZOOPS) approach can be used. In this approach, some sequences are omitted from the motif discovery if they are too dissimilar. Finally, "two-component-mixture" (TCM) allows the discovery of several non-overlapping motifs in DNA sequences.

Putative binding sites of the regulator are detected by passing known binding sites of the TF along with a stretch of 400 base pairs upstream of the start site of the presumed target gene to *cosmo*. An analysis shows that 93% of the known TFBSs of *E. coli* lie within 400 base pairs of the next gene. Since the genes of *E. coli* are organized within operons, the upstream region is chosen in front of the first gene of the corresponding operon. Even though there are cases in which TFs bind within the coding region of the corresponding operons, *e.g.,* LacI in the *lac* operon [8], only few such cases have been found.

Since TFBSs are usually not very well preserved, the ZOOPS model will be used to detect motifs in the promoter region of the presumed target gene. Hence, if a known binding site is too dissimilar to other binding sites, it will be omitted from the analysis. Furthermore, it can be detected whether the sequence of the upstream region of the presumed target gene is too dissimilar to the known binding sites. The TCM model which allows to detect multiple binding sites has not been used since it usually returned a large number of potential binding sites in the presumed target sequences (data not shown). The sequences of the known binding sites passed to *cosmo* included the binding sites and a stretch of four base pairs to either side. This is necessary since the extra stretches might facilitate the binding of the transcription factor. The maximum width of the motif had to be specified. Such the minimal length of one of the given sequences but a maximum of 30 bp is given. This is necessary since the maximal length of a motif that can be detected by *cosmo* is 30 bp. An analysis showed that only 60 (0.5%) of the known binding sites in the genome of *E.*

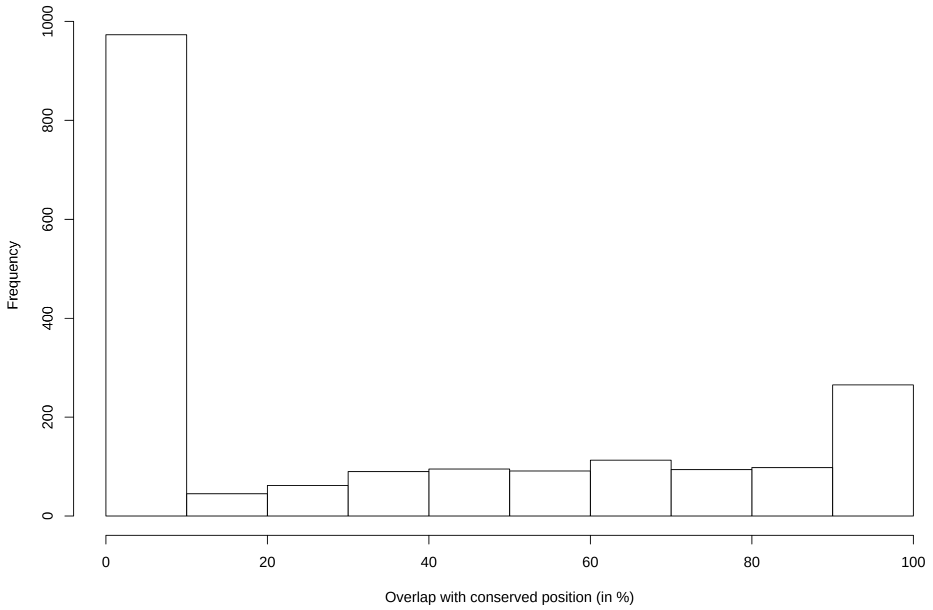

Figure 2: Overlap of known binding sites in *E. coli* with conserved positions found by [10].

coli are longer than 30 base pairs.

A simpler approach to detect motifs is the usage of position weight matrices indicating the appearance of each nucleotide on each position of several known binding sites. However, this approach would limit the analysis to 80 TFs for which position weight matrices are available from RegulonDB (`regulondb.ccg.unam.mx`, [9]). By using the approach outlined above, it is possible to examine the potential interactions of 128 TFs possessing at least one known binding site.

Phylogenetical conservation of binding sites

Comparing all known TFBSs obtained from RegulonDB to the conserved positions detected by [10] we found that 35.4% of the known binding sites of *E. coli* overlap to an extent of more than 50% with a conserved upstream region (Fig. 2). Only 9.2% lie completely within such a region. Hence, if a true interaction with the correct binding site is found it might be discarded because it does not lie within a conserved region. Nevertheless, as demonstrated in the main document, this process significantly enriches the set of inferred interactions with known TF - gene interactions.

S5 Inferring directed interactions

In contrast to mutual information, DTI allows to assign a direction to an inferred interaction. Using mutual information, in contrast, a direction needs to be inferred by additional information. Such information could be, for instance, that one partner of an interaction is a transcription factor and the other not. Thus, it can be deduced that the transcription factor exerts a regulatory effect on the gene. Using directed information this knowledge is, in principle, not required. To access whether this property of DTI can be used for the inference of directed interactions, we performed

a second analysis in which we computed the DTIs between any pair of genes. Thus, we inferred influences between the expression values of arbitrary sets of genes. Subsequently, we followed the outlined procedure and computed z-scores for each influence. Analyzing the best scoring influences we found that they mostly belonged to genes within the same operon. Furthermore, for most such influences the significance of the DTI of the forward direction is very close to the significance of the DTI of the backward direction (i.e., for two such genes i an j, the z-scores of $I(i \to j)$ and $I(j \to i)$ are very similar). Thus, we concentrated on influences for which we found a significant difference between the significance of the DTI of the forward and the backward direction (Fig. 3). Surprisingly, the resulting network which contained 156 influences between 184 genes does not contain any known TF - gene interaction. Together with the network of TF - gene interactions inferred in the main document this indicates that TF - gene interactions are only visible as co-expression within the data used. Thus, the time-scale of 30 minutes of the data we used might not be sufficient to detect a timed relationship between the change of transcription of a TF and the change of transcription of a target gene.

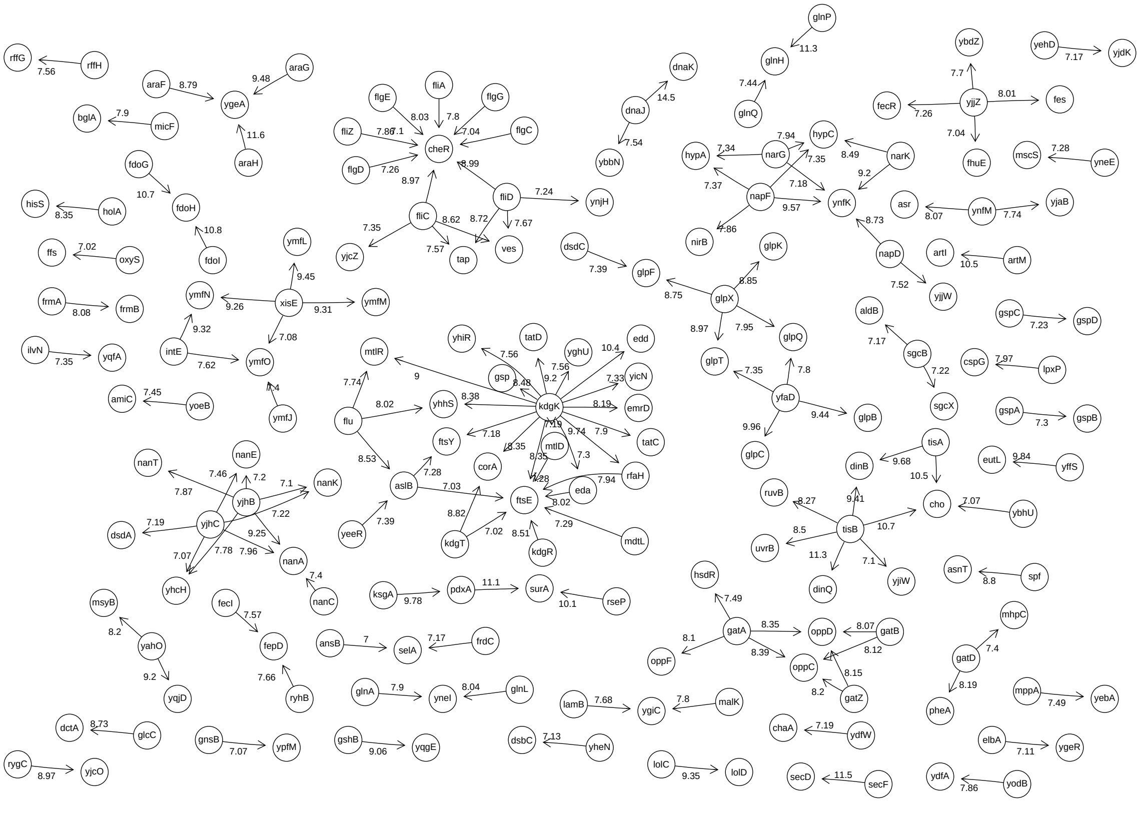

Figure 3: Influences were inferred for a z-score greater 7.0 and a differences of the significances of the forward and backward direction greater 1.5. Nodes correspond to genes and inferred influences to edges. The corresponding z-scores are indicated.

Analyzing the influences inferred for the genome-wide network we found that several influences, especially among genes of flagellar synthesis, correspond to known timed patterns in gene expression. [11] found that the genes in this pathway are expressed in three phases corresponding to the part of the structure of the flagellum assembled. The subnetwork affecting genes of flagellar assembly pathways contains 13 genes and 14 influences (Fig. 4). Analyzing the influences between

the genes and assigning them to the particular phases we found that this network reflects the known temporal patterns. Except for 3 genes not yet characterized in their function, all influences start from genes assigned to phase 2 and lead to genes belonging to phase 3. However, we could not detect any further influence corresponding to other known patterns in the timing of expression which occur, for instance, in the synthesis of amino acids [12].

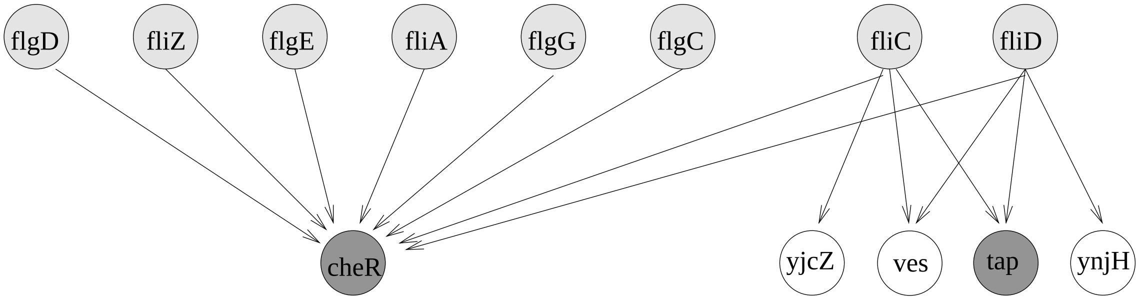

Figure 4: Nodes shaded in light gray correspond to genes transcribed in the second phase of flagellar synthesis according to [11]. Nodes corresponding to genes transcribed in the third phase are shaded in dark gray. For nodes left white, the function of the protein encoded by the corresponding gene is not yet clear.

S6 Transcription factors

In the following a list of 316 genes coding for predicted and known TFs from RegulonDB is given.

AaeR, AbgR, AcrR, Ada, AdiY, AgaR, AlaS, AllR, AllS, AlpA, AppY, AraC, ArcA, ArgP, ArgR, ArsR, AscG, AslB, AsnC, AstA, AtoC, BaeR, BasR, BetI, BglJ, BirA, BolA, CadC, CaiF, Cbl, CdaR, ChbR, ChpA, ChpB, CpxR, CreB, Crl, Crp, CsgD, CsiR, CspA, CspB, CspC, CspD, CspE, CspF, CspG, CspH, CspI, CueR, CusR, CynR, CysB, CytR, DctR, DcuR, DeoR, DgoR, DgsA, DhaR, DiaA, DicA, DnaA, DpiA, DsdC, EbgR, EnvR, EnvY, EutR, EvgA, ExuR, FabR, FadR, FeaR, FhlA, FimZ, Fis, FlhC, FlhD, Fnr, FrlR, FruR, FrvR, FucR, Fur, GadE, GadW, GadX, GalR, GalS, GcvA, GcvR, GlcC, GlnG, GlpR, GntR, GutM, HcaR, HdfR, HipA, HipB, Hns, HupA, HupB, HycA, HyfR, HypA, HypF, IclR, IdnR, IhfA, IhfB, IlvY, IscR, KdgR, KdpE, KdsD, LacI, LeuO, LexA, LldR, LpcA, LrhA, Lrp, LsrR, LysR, MalI, MalT, MarA, MarR, MatA, MelR, MetJ, MetR, MhpR, MlrA, MltD, MngR, MntR, ModE, MprA, MtlR, Nac, NadR, NagC, NanR, NarL, NarP, NhaR, NikR, NorR, NrdR, NsrR, OgrK, OmpR, OsmE, OxyR, PaaX, PdhR, PepA, PerR, PhnF, PhoB, PhoP, PhoU, PptA, PrpD, PrpR, PspC, PspF, PurR, PutA, PuuR, QseB, RacR, RbsR, RcnR, RcsA, RcsB, RfaH, RhaR, RhaS, Rob, RpiR, Rsd, RstA, RtcR, RutR, SdiA, SfsA, SfsB, SgcR, SgrR, SlmA, SlyA, SoxR, SoxS, SrlR, StpA, TdcA, TdcR, TorR, TreR, TrpR, TtdR, TyrR, UhpA, UidR, UlaR, UvrY, UxuR, WrbA, XapR, XylR, YafC, YafY, YagI, YahA, YahB, YaiV, YaiW, YbaO, YbaQ, YbcM, YbdO, YbeF, YbhD, YbhN, YbiH, YbjJ, YbjK, YbjN, YcaL, YcaN, YceN, YcfQ, YcgE, YciT, YcjW, YcjZ, YdaS, YdcI, YdcN, YdcQ, YdcR, YddM, YdeM, YdeO, YdfH, YdhB, YdhM, YdiP, YdjF, YeaM, YeaT, YebK, YedW, YeeY, YegW, YeiE, YeiL, YfaX, YfcJ, YfeC, YfeD, YfeR, YfeT, YfhA, YfhH, YfiE, YfjR, YgaV, YgbI, YgeH, YgeK, YgeR, YgeV, YgfI, YggD, YggG, YghO, YgiT, YhaJ, YheN, YheO, YhjB, YhjC, YiaJ, YiaU, YidF, YidL, YidP, YidZ, YieP, YihL, YihW, YijO, YjhI, YjhU, YjiE, YjiR, YjjJ, YjjM, YjjQ, YkgA, YkgD, YncC, YneJ, YnfL, YpdC, YqeI, YqhC, YrbA, ZntR, ZraR, Zur

S7 Interactions validated through phylogenetical conservation of binding sites

List of the 109 accepted interactions following the search for phylogenetically conserved binding sites. Column three through nine give the position of the found binding site on the genome and the overlap of a phylogenetically conserved promoter region with this binding site. Strand=1 indicates the sense strand and strand=-1 indicates the anti-sense strand of the chromosome. The position of the overlap with a phylogenetically conserved binding site is indicated in columns eight and nine. Column ten gives the z-score of the DTI. An asterisk behind the target gene denotes a known interaction according to RegulonDB. Interactions are grouped into operons. For each operon the binding site is given only once.

**Interaction**		**Presumed Binding Site**							**z-Score**
		**Position**				**Conservation**			
**Regulator**	**Target**	**Start**	**End**	**Strand**	**Length**	**%**	**Start**	**End**	
Crp	*yidQ*	3865467	3865495	1	14	58.3	3865467	3865480	8.11
DnaA	*mnmG*	3925136	3925148	-1	11	55	3925138	3925148	8.12
EnvY	*ppdD*	117566	117595	-1	17	100	117573	117589	6.55
FabR	*crp*	3483869	3483892	1	9	52.9	3483869	3483877	6.34
Fis	*dusB**	3408218	3408237	1	19	90.5	3408218	3408236	9.84
Fis	*rplM*	3376701	3376720	-1	18	100	3376702	3376719	6.2
	rpsI								6.6
FlhC	*flgB**	1130164	1130182	1	17	77.3	1130166	1130182	7.27
	*flgC**								7.12
	*flgD**								7.23
	*flgE**								6.58
	*flgF**								7.46
	*flgG**								7.05
	*flgH**								7.65
	*flgI**								7.89
	*flgJ**								7.84
FlhC	*flhA**	1964297	1964311	-1	11	61.1	1964297	1964307	7.63
	*flhB**								7.63
	*flhE**								8.11
FlhC	*fliL**	2017559	2017576	1	12	66.7	2017559	2017570	6.72
	*fliM**								7.36
	*fliN**								7.07
	*fliO**								7.28
	*fliP**								7.84
	*fliQ**								7.9
	*fliR**								6.75
FlhC	*yecR**	1986171	1986189	1	12	54.5	1986178	1986189	8.23
FlhD	*flgB**	1130164	1130182	1	17	77.3	1130166	1130182	6.82
	*flgC**								6.92
	*flgD**								7.11
	*flgE**								6.6
	*flgF**								7.43
	*flgG**								7.05
	*flgH**								7.27
	*flgI**								7.5
	*flgJ**								7.35

Interaction		Presumed Binding Site							z-Score
		Position			Conservation				
**Regulator**	**Target**	**Start**	**End**	**Strand**	**Length**	**%**	**Start**	**End**	
FlhD	*flhA**	1964297	1964311	-1	11	61.1	1964297	1964307	7.7
	*flhB**								7.44
	*flhE**								8.28
FlhD	*fliL**	2017559	2017576	1	12	66.7	2017559	2017570	6.67
	*fliM**								7.19
	*fliN**								7.19
	*fliO**								7.02
	*fliP**								7.53
	*fliQ**								7.57
	*fliR**								6.98
FlhD	*yecR**	1986171	1986189	1	12	54.5	1986178	1986189	7.32
GalS	*fadI*	2458581	2458603	-1	23	95.8	2458581	2458603	6.75
GlcC	*fadA*	4029014	4029034	-1	15	88.2	4029020	4029034	6.84
	fadB								9.12
HcaR	*feaR*	1445476	1445493	-1	17	100	1445477	1445493	6.55
IscR	*iscA**	2660272	2660287	-1	14	60.9	2660272	2660285	10.7
	*iscS**								11.4
	*iscU**								11.0
KdgR	*eda**	1932798	1932825	-1	13	59.1	1932798	1932810	8.52
	edd								8.92
KdgR	*ftsE*	3602342	3602369	-1	15	79	3602342	3602356	7.93
	ftsY								6.47
KdgR	*mtlR*	3770195	3770222	1	19	79.2	3770195	3770213	6.77
LacI	*mhpC*	367786	367811	1	19	79.2	367786	367804	8.15
	mhpD								6.77
	mhpF								6.78
LexA	*cho*	1821505	1821527	1	18	100	1821507	1821524	9.45
LexA	*dinB*	250863	250885	1	18	100	250865	250882	8.38
LexA	*dinG**	832258	832280	1	23	95.8	832258	832280	8.04
LexA	*dinI*	1120728	1120750	-1	18	100	1120731	1120748	9.84
LexA	*ftsK**	932350	932372	1	23	95.8	932350	932372	6.26
LexA	*dinF**	4255090	4255112	1	16	100	4255093	4255108	10.6
LexA	*polB**	65832	65854	-1	23	95.8	65832	65854	6.76
LexA	*recA**	2821849	2821871	-1	22	100	2821850	2821871	7.06
	*recX**								8.23
LexA	*recN**	2749748	2749770	1	22	100	2749748	2749769	9.45
LexA	*ruvA**	1944048	1944070	-1	18	100	1944051	1944068	8.97
	*ruvB**								7.3
LexA	*sulA**	1020162	1020187	-1	20	100	1020165	1020184	9.16
LexA	*umuC**	1229950	1229972	1	20	100	1229951	1229970	8.35
	*umuD**								8.55
LexA	*uvrB**	812654	812676	1	14	82.4	812663	812676	7.1
LexA	*ydjM**	1808200	1808222	1	23	95.8	1808200	1808222	8.16
LexA	*yebF*	1928787	1928809	-1	16	100	1928791	1928806	7.26
LexA	*yebG*	1928787	1928809	-1	16	100	1928791	1928806	10.4
MarR	*marA**	1617117	1617143	1	22	100	1617122	1617143	7.85
	*marB**								6.37
MelR	*yadI*	144488	144504	1	17	77.3	144488	144504	6.2
MetR	*yigM*	4008935	4008952	1	18	75	4008935	4008952	6.94
MhpR	*argT*	2425972	2425995	-1	10	52.6	2425986	2425995	10.5
NagC	*ypdA*	2496500	2496528	1	19	100	2496503	2496521	6.6

Interaction		Presumed Binding Site							z-Score
		Position			Conservation				
Regulator	Target	Start	End	Strand	Length	%	Start	End	
NhaR	*nhaA**	17386	17401	1	16	66.7	17386	17401	11.4
NikR	*ccmC*	2301815	2301844	-1	18	100	2301820	2301837	6.35
	ccmD								6.35
	ccmE								6.53
	napB								6.62
	napC								6.4
	napH								7.28
PdhR	*lipA*	659445	659468	-1	21	87.5	659448	659468	9.42
PdhR	*aceE**	122041	122064	1	17	100	122044	122060	7.38
PhoB	*phoR**	416292	416314	1	21	95.5	416294	416314	6.25
PhoP	*cysB*	1331758	1331780	1	16	76.2	1331765	1331780	7.58
PhoP	*phoQ**	1189727	1189749	-1	14	73.7	1189727	1189740	7.35
PrpR	*ugpB*	3590409	3590434	-1	16	69.6	3590409	3590424	7.4
RcsA	*flhA*	1964232	1964249	-1	11	57.9	1964239	1964249	8.24
	flhB								7.77
	flhE								7.79
RcsA	*yecR*	1985950	1985964	1	15	71.4	1985950	1985964	6.95
RcsB	*edd*	1932762	1932779	-1	18	75	1932762	1932779	6.23
RhaR	*yigM*	4009037	4009060	1	14	82.4	4009037	4009050	6.52
RhaS	*yigM*	4008967	4008987	1	21	91.3	4008967	4008987	6.32
SoxS	*yggL*	3100911	3100921	-1	11	57.9	3100911	3100921	6.29
TdcA	*dcuB*	4346779	4346798	-1	16	80	4346783	4346798	6.55

S8 Interactions validated through chromosomal position of the target gene

Additionally to the interactions reported in the main document we identified several cases in which the target lies directly downstream of an operon known to be regulated by the effector.

S8.1 Targets of IscR

From a high z-score we identified *fdx*, *hcsA*, *hcsB* and *iscX* as new targets of IscR. These four genes are involved in the formation of iron-sulfur clusters [13, 14]. IscR itself is involved in the iron-sulfur cluster biogenesis by regulating the *iscRSUA* operon which is situated directly upstream of the *hcsBA-fdx-iscX* operon. There is no agreement about whether both operons are independent or belong together since a transcription initiation site has been detected upstream of *hcsB* [15] and *hcsA* [16]. However, other work suggests a co-transcription of both operons [13, 14, 17]. The high z-score indicates that both transcription units indeed belong to the same operon or are at least tightly co-regulated by IscR.

S8.2 Targets of PaaX

From a high z-score we identified *paaY* as a new target of PaaX. Even though *paaY* is not yet characterized, a role in phenylacetate degradation has been suggested [18]. Owing to a high z-score and the regulation of the *paaABCDEFGHIJK* operon which lies directly upstream of the *paaXY* operon by PaaX, both operons might be co-transcribed.

S8.3 Additional targets of LexA

Three additional inferred targets of LexA for which no phylogenetically conserved binding site was detected are *yafN*, *yafO* and *yafP*. In these cases, additional evidence comes from their chromosomal position since they are preceded by the *dinB* gene which is a known target of LexA. Indeed all three genes code for proteins that have a presumed role in mutagenesis and are suggested to belong to an operon additionally containing *dinB* [19]. Thus, the alternative operon structure reported in [19] is further substantiated by our findings.

Furthermore, two additional predicted targets of LexA, *YbiB* and *yebF*, lie upstream of *dinG* and *yebG*, respectively, both known targets of LexA [20]. Even though both genes have not yet been characterized according to EcoCyc [21], in conjunction with gene expression analysis, it can be argued that these genes form operons together with the preceding genes and thus *ybiB* and *yebF* are further targets of LexA.

S8.4 Additional targets of PdhR

For the inferred interaction between PdhR and *ycfJ*, we found additional evidence in the chromosomal position of *ycfJ*. *YcfJ* is situated directly downstream of *ndh*, a gene known to be regulated by PdhR [22]. However, the protein encoded by this gene is not yet completely characterized even though it was found that a *ycfJ*- mutant is impaired in biofilm formation [23].

S9 Additional interactions reported in the literature

Comparing the binding sites of Fis found by [24] and the targets of Fis predicted by a significant DTI we identified *ycaO* as an additional correctly predicted TF - gene interaction.

References

1. Rao, A, Hero, AO, States, DJ, Engel, JD (2007) Using directed information to build biologically relevant influence networks. Comput Syst Bioinformatics Conf 6: 145–156.

2. Faith, JJ, Hayete, B, Thaden, JT, Mogno, I, Wierzbowski, J, et al. (2007) Large-scale mapping and validation of *Escherichia coli* transcriptional regulation from a compendium of expression profiles. PLoS Biol 5: e8.

3. Daub, CO, Steuer, R, Selbig, J, Kloska, S (2004) Estimating mutual information using B-spline functions–an improved similarity measure for analysing gene expression data. BMC Bioinformatics 5: 118.

4. Basso, K, Margolin, AA, Stolovitzky, G, Klein, U, Dalla-Favera, R, et al. (2005) Reverse engineering of regulatory networks in human B cells. Nat Genet 37: 382–390.

5. Margolin, AA, Nemenman, I, Basso, K, Wiggins, C, Stolovitzky, G, et al. (2006) ARACNE: an algorithm for the reconstruction of gene regulatory networks in a mammalian cellular context. BMC Bioinformatics 7 Suppl 1: S7.

6. Bembom, O, Keles, S, van der Laan, MJ (2007) Supervised detection of conserved motifs in DNA sequences with cosmo. Stat Appl Genet Mol Biol 6: Article8.

7. Rudd, KE (2000) EcoGene: a genome sequence database for *Escherichia coli* K-12. Nucleic Acids Res 28: 60–64.

8. Lewis, M (2005) The lac repressor. C R Biol 328: 521–548.

9. Gama-Castro, S, Jimínez-Jacinto, V, Peralta-Gil, M, Santos-Zavaleta, A, naloza Spinola, MIP, et al. (2008) RegulonDB (version 6.0): gene regulation model of *Escherichia coli* K-12 beyond transcription, active (experimental) annotated promoters and Textpresso navigation. Nucleic Acids Res 36: D120–D124.

10. McCue, L, Thompson, W, Carmack, C, Ryan, MP, Liu, JS, et al. (2001) Phylogenetic footprinting of transcription factor binding sites in proteobacterial genomes. Nucleic Acids Res 29: 774–782.

11. Kalir, S, McClure, J, Pabbaraju, K, Southward, C, Ronen, M, et al. (2001) Ordering genes in a flagella pathway by analysis of expression kinetics from living bacteria. Science 292: 2080–2083.

12. Zaslaver, A, Mayo, AE, Rosenberg, R, Bashkin, P, Sberro, H, et al. (2004) Just-in-time transcription program in metabolic pathways. Nat Genet 36: 486–491.

13. Takahashi, Y, Nakamura, M (1999) Functional assignment of the *ORF2-iscS-iscU-iscA-hscB-hscA-fdx-ORF*3 gene cluster involved in the assembly of Fe-S clusters in *Escherichia coli.* J Biochem 126: 917–926.

14. Tokumoto, U, Takahashi, Y (2001) Genetic analysis of the *isc* operon in *Escherichia coli* involved in the biogenesis of cellular iron-sulfur proteins. J Biochem 130: 63–71.

15. Lelivelt, MJ, Kawula, TH (1995) Hsc66, an Hsp70 homolog in *Escherichia coli*, is induced by cold shock but not by heat shock. J Bacteriol 177: 4900–4907.

16. Seaton, BL, Vickery, LE (1994) A gene encoding a DnaK/hsp70 homolog in *Escherichia coli.* Proc Natl Acad Sci U S A 91: 2066–2070.

17. Giel, JL, Rodionov, D, Liu, M, Blattner, FR, Kiley, PJ (2006) IscR-dependent gene expression links iron-sulphur cluster assembly to the control of O2-regulated genes in *Escherichia coli.* Mol Microbiol 60: 1058–1075.

18. Ferrández, A, nambres, BM, García, B, Olivera, ER, Luengo, JM, et al. (1998) Catabolism of phenylacetic acid in *Escherichia coli.* Characterization of a new aerobic hybrid pathway. J Biol Chem 273: 25974–25986.

19. McKenzie, GJ, Magner, DB, Lee, PL, Rosenberg, SM (2003) The *dinB* operon and spontaneous mutation in *Escherichia coli.* J Bacteriol 185: 3972–3977.

20. Wade, JT, Reppas, NB, Church, GM, Struhl, K (2005) Genomic analysis of LexA binding reveals the permissive nature of the *Escherichia coli* genome and identifies unconventional target sites. Genes Dev 19: 2619–2630.

21. Keseler, IM, Collado-Vides, J, Gama-Castro, S, Ingraham, J, Paley, S, et al. (2005) EcoCyc: a comprehensive database resource for *Escherichia coli.* Nucleic Acids Res 33: D334–D337.

22. Ogasawara, H, Ishida, Y, Yamada, K, Yamamoto, K, Ishihama, A (2007) PdhR (pyruvate dehydrogenase complex regulator) controls the respiratory electron transport system in *Escherichia coli.* J Bacteriol 189: 5534–5541.

23. Beloin, C, Valle, J, Latour-Lambert, P, Faure, P, Kzreminski, M, et al. (2004) Global impact of mature biofilm lifestyle on *Escherichia coli* K-12 gene expression. Mol Microbiol 51: 659–674.

24. Cho, BK, Knight, EM, Barrett, CL, Palsson, BØ (2008) Genome-wide analysis of Fis binding in *Escherichia coli* indicates a causative role for A-/AT-tracts. Genome Res 18: 900–910.
